# Metabotropic glutamate receptor 5 in bulimia nervosa

**DOI:** 10.1038/s41598-020-63389-7

**Published:** 2020-04-14

**Authors:** Yoan Mihov, Valerie Treyer, Funda Akkus, Erika Toman, Gabriella Milos, Simon M. Ametamey, Anass Johayem, Gregor Hasler

**Affiliations:** 1 Translational Research Center, University Hospital of Psychiatry and Psychotherapy, University of Bern, Bern, Switzerland; 2 Departement of Nuclear Medicine, University Hospital Zürich, University of Zürich, 8091 Zürich, Switzerland; 3 Competence Network for Eating Disorders, Forchstrasse 132, 8032 Zürich, Switzerland; 4 Department of Consultation-Liaison Psychiatry and Psychosomatic Medicine, University Hospital Zürich, Culmannstrasse 8, 8091 Zürich, Switzerland; 5 Center for Radiopharmaceutical Science of ETH, PSI, and USZ, Department of Chemistry and Applied Biosciences of ETH, 8093 Zürich, Switzerland; 6 Psychiatry Research Unit, University of Fribourg, Chemin du Cardinal-Journet 3, 1752 Villars-sur-Glâne, Switzerland

**Keywords:** Positron-emission tomography, Psychiatric disorders

## Abstract

Bulimia nervosa (BN) shares central features with substance-related and addictive disorders. The metabotropic glutamate receptor subtype 5 (mGlu5) plays an important role in addiction. Based on similarities between binge eating and substance-related and addictive disorders, we investigated mGlu5 *in vivo* in 15 female subjects with BN and 15 matched controls. We measured mGlu5 distribution volume ratio (DVR) with positron emission tomography (PET) using [11 C]ABP688. In BN mGlu5 DVR was higher in the anterior cingulate cortex (ACC), subgenual prefrontal cortex, and straight gyrus (p < 0.05). In BN, higher mGlu5 DVR in various brain regions, including ACC, pallidum, putamen, and caudate, positively correlated with “maturity fears” as assessed using the Eating Disorder Inventory-2 (p < 0.05). In BN and controls, smokers had globally decreased mGlu5 DVR. We present the first evidence for increased mGlu5 DVR in BN. Our findings suggest that pharmacological agents inhibiting mGlu5 might have a therapeutic potential in BN.

## Introduction

The metabotropic glutamate receptor subtype 5 (mGlu5) plays an important role in glutamate signalling. mGlu5 is a G-protein-coupled seven transmembrane protein with two binding sites and a structural connection to NMDA receptors through a chain of scaffold proteins^1,2^. Glutamate activates mGlu5 by binding to its orthosteric binding site^1^. mGlu5 modulates NMDA receptor excitability and contributes to structural changes involved in adaptive synaptic plasticity, long-term potentiation, and learning^2–4^. mGlu5 regulates glutamate-dependent developmental changes^5^. It is involved in the reinforcing action of natural stimuli, such as food, and drugs of abuse^6–8^. mGlu5 plays an important role in aberrant synaptic plasticity in addiction^8,9^. An impressive body of research demonstrates that pharmacological inhibition of mGlu5 reduces self-administration of substances of abuse in animal models of addiction^6,8,10,11^. In addition, preclinical studies demonstrate inhibited feeding behavior after application of selective negative allosteric modulators of mGlu5 (mGlu5 NAMs)^6,8^. This evidence suggests a role for mGlu5 in regulation of feeding behavior and eating disorders.

Based on the behavioral and neurobiological similarities between binge eating and substance use disorders, a food addiction model has received recognition as a new and promising research perspective^12–14^. Phenomenologically, there is a close relationship between addictive disorders and binge eating behavior, a major symptom of BN^12,15^. Using PET imaging, we and other groups have demonstrated altered mGlu5 binding in various substance use disorders, such as cocaine, alcohol, and nicotine addiction^16–23^.

The preclinical evidence for mGlu5 in feeding behavior and its important role in addictive behaviors in general suggest a role for mGlu5 in BN. To investigate mGlu5 in BN we carried out a PET study with the selective mGlu5 tracer 3‐(6‐methyl‐pyridin‐2‐ylethynyl)‐cyclohex‐2‐enone‐O‐11 C‐methyl‐oxime ([11 C]ABP688) in individuals with BN and healthy controls^24^. We determined mGlu5 DVR for 35 brain regions, using the cerebellum as a reference region. We previously showed that the cerebellum is a suitable reference region due to its low mGlu5 levels^24–26^. We compared mGlu5 DVR between both diagnostic groups in each brain region and accounted for the effects of smoking. We tested whether correlation patterns of mGlu5 DVR across brain regions differ between both diagnostic groups^17^. Finally, we analysed the relationship between mGlu5 DVR and clinical characteristics.

## Results

Individuals with BN and controls did not differ in age or body mass index (BMI), as indicated by two-tailed Welch’s tests (p > 0.05, Table 1). BN individuals had higher BAI and BDI scores than controls (p < 0.05, two-tailed, Table 1). In the BN group, 1 subject fulfilled the diagnostic criteria of major depressive episode, 1 of social phobia, 1 of post-traumatic stress disorder (PTSD), and 1 of insecure/avoidant personality disorder.

**Table 1:** Demographic and clinical characteristics of the study samples.

	BN (n = 15)		HC (n = 15)		Difference
	M ± SD	Range	M ± SD	Range	p-value
Age	29.3 ± 7.1	20–44	28.9 ± 6.7	18–44	0.9
Number of current Smokers	5		5		—
Number of short-term ex-smokers	1		1		—
Number of long-term ex-smokers	1		1		—
Number of non-smokers	8		8		—
BDI	18.9 ± 11.6	3–35	1.8 ± 1.7	0–5	<0.01
BAI	13.3 ± 9.7	2–31	4.4 ± 4.1	0–12	<0.01
Age of onset	19.7 ± 5.7	13–32	—	—	—
Duration of illness (yrs)	8.4 ± 5.6	1–20	—	—	—
Imipramine Equivalent	128.6 ± 120.9	0–405	—	—	—
BMI	21.6 ± 3.8	18–30.8	22.9 ± 3.3	19.2–29.7	0.32

***M ± SD*** refers to mean ± standard deviation, ***Range*** refers to the most extreme values in the sample: minimum - maximum, ***p-value*** refers to 2-tailed p-values from Welch’s tests, uncorrected for multiple comparisons. ***Smokers***, ***short-term ex-smokers***, ***long-term ex-smokers***, and ***non-smokers***, as defined previously^18^, ***BDI*** refers to Beck Depression Inventory, ***BAI*** refers to Beck Anxiety Inventory, ***Age of onset*** refers to the age of onset of bulimia nervosa in years, ***Duration of illness*** refers to the duration of bulimia nervosa in years, ***Imipramine equivalent*** refers to the medication imipramine equivalent, ***BMI*** refers to body mass index.

**Figure 1:**
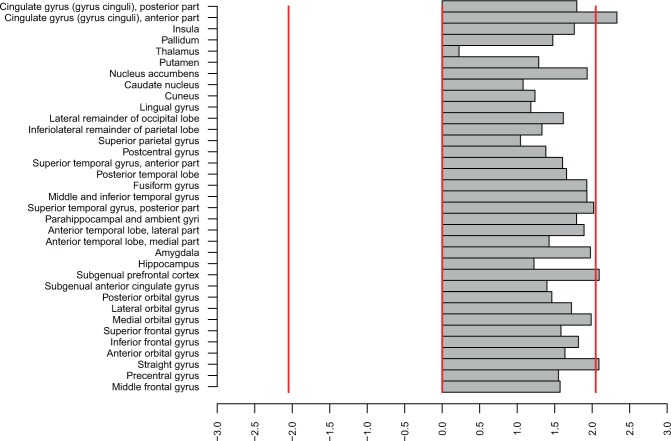
Differences between BN and HC in mGlu5 DVR. The figure shows the distribution of t-values for the comparisons between BN and HC with respect to mGlu5 DVR. Brain regions are listed on the left side of the figure, along the y-axis. For each brain region, a horizontal grey bar shows the t-value for the comparison of BN vs HC (n = 15 per group). The x-axis shows a scale for t-values. For illustration purposes, two vertical red lines at t = −2.0484 and t = 2.0484 show significance thresholds corresponding to p = 0.05, 2-tailed, based on a t-distribution with 28 degrees of freedom. The vertical red line corresponds to t = 0. Thus, grey bars to the right of the central red line correspond to positive t-values and indicate that mGlu5 DVR of BN > HC for the corresponding brain region. Conversely, a grey bar to the left of the central red line corresponds to a negative t-value and indicates that mGlu5 DVR of BN < HC for the corresponding brain region. The lengths of the grey bars indicate the magnitude of the difference and the respective t-values, from 2-tailed Welch’s tests. The three grey bars crossing the significance threshold indicate three brain regions for which mGlu5 in BN > HC: subgenual prefrontal cortex (sgPFC) (p = 0.046, t = 2.093), straight gyrus (p = 0.046, t = 2.09), and anterior cingulate gyrus (ACC) (p = 0.027, t = 2.33) (all p-values from 2-tailed Welch’s tests, uncorrected for multiple comparisons).

**Figure 2:**
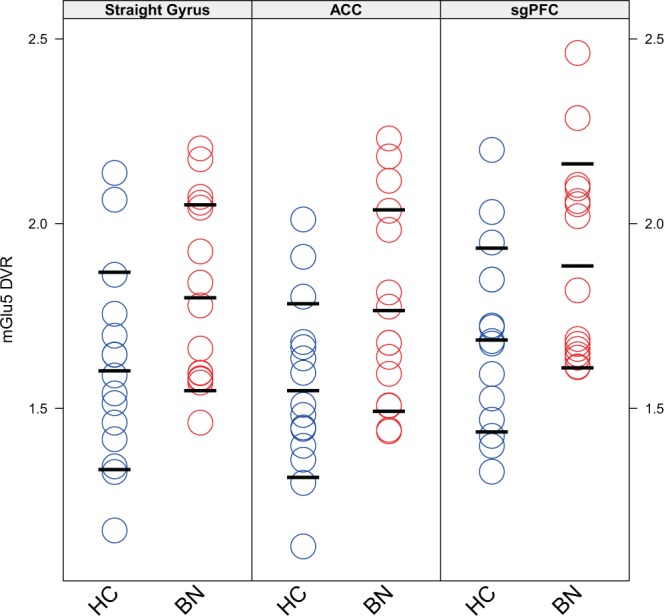
mGlu5 DVR in the straight gyrus, the anterior cingulate cortex, and the subgenual prefrontal cortex. The figure shows the distribution of mGlu5 DVR in three brain regions. Data is shown for three brain regions: the straight gyrus (***Straight Gyrus***), the anterior cingulate cortex (***ACC***), and the subgenual prefrontal cortex (***sgPFC***). mGlu5 DVR-values are shown on the y-axis. Circles represent individual data points, blue indicates healthy controls (***HC***, n = 15), red indicates bulimia nervosa (***BN***, n = 15). For each brain region and each group, three horizontal black lines show the mean ± one standard deviation of mGlu5 DVR-values.

**Figure 3:**
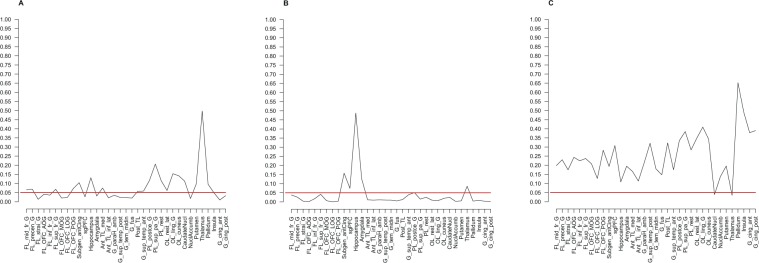
The effects of diagnostic group, smoking status, and their interaction on mGlu5 DVR. The figure summarizes the results of 35 ANOVAs, fitting a main effect of group (HC vs. BN, n = 15 per group), a main effect of smoking (smokers vs. non-smokers), and an interaction effect group*smoking on mGlu5 DVR for 35 brain regions. ***Panel a*** shows the results for the main effect of group, with brain regions along the x-axis and p-values on the y-axis. A broken black line connects the p-values corresponding to the main effect of group for each brain region. A straight horizontal red line indicates the significance threshold (p = 0.05, uncorrected for multiple comparisons). Dips of the black line below the significance threshold indicate higher mGlu5 DVR in BN than in HC for the corresponding brain region (p < 0.05, uncorrected for multiple comparisons). ***Panel b*** shows a summary for the main effects of smoking, notation analogous to Panel a. Significant main effect of smoking corresponds to lower mGlu5 DVR in smokers than in non-smokers (p < 0.05, uncorrected for multiple comparisons). ***Panel c*** shows a summary for the interaction effect group*smoking with notation analogous to Panel a.

Direct comparisons between mGlu5 DVR in subjects with BN and healthy controls for all brain regions are shown in Fig. 1. A series of Welch’s tests showed significant differences in 3 contiguous regions: ACC, the straight gyrus, and sgPFC, a small region corresponding to the pre-subgenual anterior cingulate gyrus in the anatomical maximum probability atlas of our PET analysis software (see Methods below) (Supplementary Table S3, Fig. 2, p < 0.05, two-tailed, uncorrected for multiple comparisons). These results were corroborated and extended by a series of ANOVAs that, in addition, accounted for the variance explained by smoking (Fig. 3). These ANOVAs confirmed the effect of diagnostic group on mGlu5 DVR in the ACC, the sgPFC, and the straight gyrus (p < 0.05). Moreover, the ANOVAs showed significant effect of diagnostic group on mGlu5 DVR for the following subcortical, frontal, and temporal brain regions (Fig. 3a, p < 0.05, uncorrected for multiple comparisons): the nucleus accumbens, the medial, anterior, and lateral orbital gyrus, the inferior frontal gyrus, the amygdala, the insula, the fusiform gyrus, the middle and inferior temporal gyrus, the posterior part of the superior temporal gyrus, the parahippocampal and ambient gyri, the inferior lateral part of the anterior temporal lobe, and the posterior cingulate gyrus. A significant main effect of smoking indicated decreased mGlu5 DVR in smokers, in accordance with our previous findings (Fig. 3b, p < 0.05, uncorrected for multiple comparisons)^16,18,27^. We found a significant diagnostic group-by-smoking interaction in the caudate nucleus and the thalamus (Fig. 3c, p < 0.05, uncorrected for multiple comparisons). This interaction indicated that in both regions mGlu5 DVR was higher in BN than in HC in smokers, but not in non-smokers. Overall, the results showed increased mGlu5 DVR in BN in frontal and temporal cortical and subcortical brain regions.

**Figure 4:**
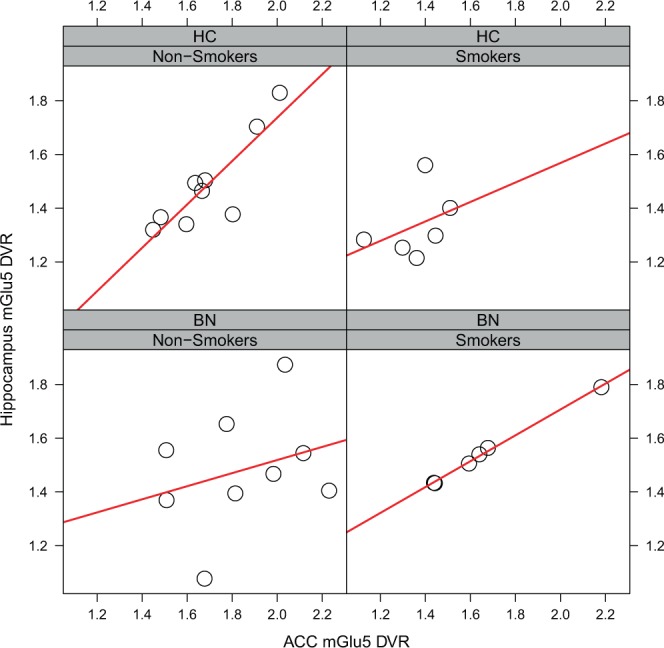
Hippocampus-ACC mGlu5 DVR-correlations in subjects with bulimia nervosa and healthy controls. The figure shows the relation between Hippocampus mGlu5 DVR (y-axis) and ACC mGlu5 DVR (x-axis) in healthy controls (upper row) and subjects with bulimia nervosa (lower row). Each diagnostic group is subdivided into non-smokers (left column, n = 9) and smokers (right column, n = 6). Pearson’s r correlation between hippocampus and ACC mGlu5 was significantly higher in smokers with bulimia than in healthy smokers (smokers with bulimia: Pearson’s r = 0.998, healthy smokers: Pearson’s r = 0.382, difference p-value < 0.001, two-tailed, uncorrected for multiple comparisons). This difference was not significant for the comparison between non-smokers with bulimia and healthy non-smokers (non-smokers with bulimia: Pearson’s r = 0.289, healthy non-smokers: Pearson’s r = 0.867, difference p-value = 0.077, two-tailed, uncorrected for multiple comparisons). Correlation comparisons were carried out using Fisher’s r-to-z transformation as reported previously^17^.

The explorative comparisons of mGlu5 DVR correlation matrices revealed one significant difference between subjects with BN and controls (Supplementary Figs. S1–7): the correlation between mGlu5 DVR in the ACC and the hippocampus was higher in smokers with BN than in healthy smokers (Fig. 4, smokers with bulimia: Pearson’s r = 0.998, healthy smokers: Pearson’s r = 0.382, Fisher’s r-to-z difference p-value < 0.001, two-tailed, uncorrected for multiple comparisons). We did not find the same pattern of ACC-hippocampus mGlu5 DVR correlations in non-smokers (Fig. 4, non-smokers with BN: Pearson’s r = 0.289, non-smoking healthy controls: Pearson’s r = 0.867, difference p-value = 0.077, two-tailed, uncorrected for multiple comparisons). Moreover, the pattern of inter-regional mGlu5 DVR-correlations differed markedly between smokers and non-smokers with BN and to a lesser extent between healthy smokers and healthy non-smokers (Supplementary Figs. S1 and S4 for healthy non-smokers and healthy smokers, respectively; Supplementary Figs. S2 and S5 for non-smokers and smokers with BN, respectively).

Out of 1,155 explorative correlation tests ten yielded significant results: higher scores on the EDI-2 scale “Maturity Fears” corresponded to higher, i.e., more aberrant, mGlu5 DVR in ten brain regions: ACC, pallidum, putamen, caudate nucleus, amygdala, insula, fusiform gyrus, inferior lateral part of the anterior temporal lobe, parahippocampal and ambient gyri, and thalamus (Supplementary Fig. S7, p < 0.05, two-tailed, uncorrected for multiple comparisons). We found no further significant correlations with clinical measures or demographic variables. Thus, in individuals with BN, we found no significant correlations between mGlu5 DVR and the average scores or individual scale-scores of the self-report questionnaire EDE-Q (all p > 0.05, two-tailed, uncorrected for multiple comparisons). Moreover, we found no significant correlations between mGlu5 DVR and eating disorder symptoms assessed with items 13–18 in EDE-Q (all p > 0.05, two-tailed, uncorrected for multiple comparisons). We found no significant correlations between mGlu5 DVR and sum scores in EDI-2 (all p > 0.05, two-tailed, uncorrected for multiple comparisons). We found no significant correlations between mGlu5 DVR and BDI, BAI, age, age of onset, years of education, duration of illness, imipramine equivalent, body mass index at scan, body mass index at follow-up, or the number of weeks with binge and purge episodes in the six months after following PET.

We found no significant effect of antidepressant treatment with selective serotonin reuptake inhibitors or selective serotonin-norepinephrine reuptake inhibitors (SSRIs and SSNRIs) on mGlu5 DVR in any brain region in subjects with BN. We found no significant differences in mGlu5 DVR in any brain region when restricting these comparisons to smokers with BN, to exclude a possible confounding effect of smoking (n = 3 per group, Supplementary Tables S1–S2).

At follow-up ten of the 15 subjects from the BN group fulfilled diagnostic criteria for BN, three reported being in partial remission, one stayed in full remission, one fulfilled the diagnostic criteria for anorexia nervosa. We ran an explorative correlation analysis between pooled z-transformed mGlu5 DVR and BMI and the number of weeks with combined binge and purge-episodes. These analyses yielded no significant correlations (all p-values > 0.05, uncorrected for multiple comparisons).

## Discussion

We present the first *in vivo* investigation of mGlu5 in BN. We observed the most consistent mGlu5 DVR increases in BN in three anatomically adjacent regions: ACC, sgPFC, and straight gyrus. Smokers with and without BN showed globally decreased mGlu5 DVR. We found no significant relationships between mGlu5 DVR and BN severity as assessed using total scores of EDI-2 and EDE-Q. Higher scores on the EDI-2 scale “Maturity fears”, measuring distress with the demands of adult life, were related to higher mGlu5 in various brain regions implicated in BN, such as the ACC, pallidum, amygdala, putamen, and the caudate nucleus.

Preclinical studies showed therapeutic potential for pharmacological agents targeting mGlu5 in addiction^6,8^. mGlu5 NAMs inhibit self-administration of addictive substances in rodents and monkeys. In addition, preclinical studies show that mGlu5 is involved in the reinforcing properties of food^8^, which makes it relevant for the pathophysiology and treatment of eating disorders. In particular, feeding behavior is inhibited by the application of mGlu5 NAMs^8^. This effect is only reliable at very high doses resulting in receptor occupancy rates > 80%^8^. Thus, at mid-range doses mGlu5 NAMs inhibit binge-like self-administration of addictive drugs without impairing natural feeding behavior^8^. Importantly, preclinical research suggests that mGlu5 NAMs could specifically inhibit consumption of highly palatable food, which is typically consumed during episodes of binge eating, without affecting consumption of standard food^7^. Altogether, these findings implicate mGlu5 in the regulation of feeding behavior and suggest that mGlu5 might play a role in bulimia nervosa (BN). However, long-term mGlu5 NAM treatment-induced cognitive side effects might negatively impact cognitive control and reduce the efficacy of cognitive-behavioral therapy of BN^28,29^. mGlu5 positive allosteric modulators have been demonstrated to improve cognitive functioning including extinction learning and behavioral flexibility^30^. Novel mGlu5 NAMs with better side effect profiles are promising candidates for the treatment of BN^31,32^. Silent allosteric modulators of mGlu5 appear particularly promising, as they might have therapeutic effects with less cognitive side effects^33^.

Clinical PET studies demonstrated abnormal mGlu5 binding in various psychiatric conditions. In MDD, we found decreased mGlu5 DVR in various brain regions, while in OCD and alcohol use disorder, which are frequently comorbid with BN, we found increased mGlu5 DVR^17,34,35^. The most prominent and consistent finding from human PET studies has been globally decreased mGlu5 DVR in smokers. Specifically, we and others observed decreased mGlu5 DVR in current healthy smokers and smokers with various psychiatric conditions, including schizophrenia and cocaine use disorder^16,18,19,27^. Studies in cocaine use disorder showed a less consistent tendency towards decreased mGlu5 binding^19–21^. We observed normalization in mGlu5 DVR after prolonged abstinence from smoking^18^. Clinical PET studies on alcohol use disorder are in disagreement. We found elevated mGlu5 DVR, whereas others reported decreased mGlu5 availability^17,23^. Differences between the effects of substances of abuse on mGlu5 might reflect their different pharmacodynamic profiles. Heterogeneous findings for the same substance of abuse may reflect differences in imaging methods, clinical heterogeneity of study samples^36^. Importantly, both human PET studies and animal models with different mGlu5 PET tracers and protocols showed normalization in mGlu5 after prolonged abstinence from alcohol abuse^18,22,37–39^. Taken together, these findings suggest that disease stage is relevant for the study of mGlu5 in psychiatric conditions.

In this study, we found higher mGlu5 DVR in the ACC and medial orbitofrontal cortex (mOFC) in participants with bulimia. Both regions are involved in processing emotional and cognitive information related to self-control^40–42^. In addition, both regions have been implicated in reward-guided behavior and food value representation^43–45^. Therefore, altered mGlu5 in the ACC and mOFC could contribute to BN symptoms by impairing control over food intake and weight concerns.

Clinical studies show a complex pattern of findings on the relationship between mGlu5 and depression^46^. In contrast to our previous findings, we did not find a significant relationship between depression symptoms and mGlu5 DVR in the current study^35^. Therefore, higher depression symptoms cannot explain the increase in mGlu5 DVR we observed in subjects with BN. In a previous investigation of mGlu5 in schizophrenia, we reported an interaction between smoking, medication, sex, and diagnosis^27^. Therefore, we tested the effects of antidepressant medication on mGlu5 in subjects with BN. We found no significant correlation between imipramine equivalent and mGlu5 DVR in any brain region. Furthermore, we found no significant mGlu5 DVR differences between BN subjects receiving SSRIs or SSNRIs and BN subjects off antidepressant treatment in any brain region. Overall, we found no evidence for an effect of psychotropic medication on mGlu5 DVR in this study. Our previous findings regarding the effects of antidepressant treatment on mGlu5 DVR have been mixed. In subjects with obsessive-compulsive disorder, we found no effect of antidepressant medication on mGlu5 DVR^34^. In individuals with major depressive disorder we found lower mGlu5 DVR in persons with a history of antidepressant medication, as compared to drug-naïve participants^35^. In subjects with alcohol use disorder we found that higher imipramine equivalent dose corresponds to lower mGlu5 DVR in the subgenual anterior cingulate^17^. Studies in rodents demonstrated increased mGlu5 binding after prolonged administration of escitalopram, reboxetine, or imipramine^47,48^. Given these heterogeneous findings, larger studies with longitudinal pre-post designs and larger samples are needed to evaluate the action of SSRIs and SSNRIs on mGlu5^49^.

Our exploratory analyses suggested that elevated mGlu5 DVR might be related to increased stress and anxiety levels in BN. In the ACC, we found a relationship between higher ACC mGlu5 DVR and higher scores in self-reported “Maturity Fears” as assessed using the Eating Disorder Inventory 2 (EDI-2). This scale assesses stress related to the expectations and challenges of adult life^50–52^. This preliminary finding may suggest that role expectations play a role in the relationship between ACC mGlu5 DVR and anxiety as we found no relation between general anxiety symptoms, as measured with Beck Anxiety Inventory (BAI), and mGlu5 DVR in BN. Imaging studies in clinical samples show elevated mGlu5 in stress-related and anxiety disorders^34,53,54^. Preclinical studies suggest an anxiolytic potential for negative allosteric modulators of mGlu5^53^.

We found globally decreased mGlu5 DVR in smokers with and without BN, consistent with our previous findings and independent studies^16,18,19,27^. Importantly, the increased mGlu5 DVR we observed in the ACC, sgPFC, and the straight gyrus in BN cannot be attributed to a different fraction of smokers in both diagnostic groups as they comprised the same number of current, ex-smokers, and non-smokers. We found an interaction between BN and the effect of smoking: in the caudate and thalamus we observed higher mGlu5 DVR in BN than in HC only in smokers, but not in non-smokers. This interaction was paralleled by the effect of smoking on the correlational pattern of mGlu5 in subjects with BN and controls: we only found a significant difference between mGlu5 inter-regional correlational patterns of healthy smokers and smokers in BN, but not between their non-smoking counterparts. Overall, our data indicate an interaction both at the level of average mGlu5 DVR between group-comparisons and at the level of mGlu5 inter-regional correlation pattern-comparisons. Altogether, the effects of smoking appeared less pronounced in subjects with BN. This interaction could reflect the opposing action of two neural processes on mGlu5 in BN. Chronic nicotine-induced surge in glutamate release caused by smoking might drive an adaptive global down-regulation of mGlu5^16,18^. In subjects with BN, but not in healthy controls, mGlu5 down-regulation might be compromised by increases in mGlu5 activity related to compulsive binge-eating behavior.

Several limitations merit comment. Because mGlu5 interacts with monoamine receptor systems, altered mGlu5 might contribute to BN symptoms indirectly, by impacting dopamine and serotonin signalling^13,55–59^. However, this assumption remains speculative because, in our current study we did not assess monoamine functioning. Multimodal imaging studies are needed to assess both mGlu5, monoamine functioning, and their interactions in BN. The relatively small sample sizes, the large fraction of smokers in both groups, and the clinical heterogeneity in the BN group might have influenced mGlu5 DVR and our results. Power analyses indicated that our sample size yielded statistical power of 61% for the effect we found in the anterior cingulate (Supplementary Table S3). Small effect sizes require considerably larger sample sizes to be tested with high statistical power. Small regions such as the sgPFC yield a signal that is more susceptible to noise artefacts and should be interpreted with caution. Multiple correlation tests such as carried out in our exploratory analyses increase the risk of false positive findings. Independent replications with different protocols or new specific mGlu5 tracers are needed to corroborate and extend our findings^60,61^. Importantly, larger study samples yielding higher statistical power are needed. Longitudinal investigations of subjects with BN during episodes of acute illness and after periods of sustained remission could provide further important insights, as demonstrated in nicotine addiction and alcohol use disorder^18,22,37–39^. Postmortem studies could provide alternative types of evidence for the implication of mGlu5 in BN^35^.

In conclusion, this study encourages future investigations of mGlu5 in eating disorders and related conditions such as binge eating disorder and stress-related eating in obesity. It suggests that abnormal mGlu5 signalling contributes to impaired social stress processing and self-control in a wide range of psychiatric conditions.

## Methods

### Subjects

We recruited 15 female individuals with bulimia nervosa through the Department of Psychiatry and Psychotherapy, University Hospital Zürich, the Competence Network for Eating Disorders Zürich, as well through the local print media. We included subjects fulfilling diagnostic criteria for BN according to the Diagnostic and Statistical Manual of Mental Disorders, Fifth Edition (DSM-5)^15^. At the time of recruitment, 10 fulfilled the full diagnostic criteria, 4 were in partial remission, and 1 was in full remission. Twelve of the fifteen participants with bulimia nervosa received antidepressant medication at the time of scanning. Eight received SSRIs, four SSNRIs, one received bupropion, one quetiapine, and one topiramate. Eleven subjects received SSRIs, SSNRIs, or both. Exclusion criteria comprised pregnancy, breastfeeding, neurological diseases, a history of psychosis, and current substance use disorder. Psychopathology was assessed with the Structured Clinical Interview for DSM-IV (SCID)^62^. In addition, participants filled out BAI and Beck Depression Inventory (BDI) as self-report measures of anxiety and depressive symptoms, respectively^63,64^. Eating disorder symptomatology was assessed with the Eating Disorder Examination – Questionnaire (EDE-Q) and the EDI-2^50–52,65^. We assessed the age of onset of BN, illness duration, and the imipramine equivalent of their current psychiatric medication following the method by^66^. We asked participants with BN for their agreement to call them for a follow-up assessment of eating disorder symptomatology 6 months after PET imaging. All agreed. We reached and interviewed them between six and eight months after the PET scan. At follow-up, we assessed diagnostic criteria for BN with SCID-IV. In addition, we asked subjects with BN about their current weight and the number of weeks in which they experienced binge and purge episodes during the six months following the PET scan.

We compared the BN group to a group of 15 age and smoking status-matched healthy control women (HC group). Thus, each group comprised eight non-smokers, five current smokers, one long-term ex-smoker, and one short-term ex-smoker, as defined previously^18^. The control group comprised the data from 11 individuals that had participated in previous studies^16,35^ and 4 newly recruited participants. We excluded participants with a history of psychiatric disorders. Further exclusion criteria comprised pregnancy, breastfeeding, and neurological diseases. New participants were recruited using an online platform. We used SCID to exclude psychopathology in controls. In addition, controls provided demographic data and filled out BAI and BDI.

All subjects provided informed consent prior to study participation. The study and the consent forms were approved by the local ethics committee Zürich (Kantonale Ethikkommission Zürich). The study was carried out in accordance with the guidelines of the Swiss Academy of Medical Sciences (SAMW) and applicable Swiss law.

### Positron emission tomography

We carried out PET imaging with the selective mGlu5 radiotracer [11 C]ABP688 according to an established protocol^16–18,24,27,34,35^. Briefly, participants received a low-dose CT, followed by a PET scan. [11 C]ABP688 was given intravenously in a volume of 50 ml over one hour. An infusion pump administered half of the radiotracer in the first two minutes as a bolus and the other half over the following 58 minutes. Participants received a total radioactivity dose of 600–800 MBq. To rule out circadian fluctuations in mGlu5 binding as a source of data variance, we carried out all scanning on the afternoon.

### Statistical analysis

We carried out statistical analysis in three major steps: calculation of mGlu5 distribution volume ratio (DVR), mGlu5 DVR group comparisons, and explorative analyses on the relationship between mGlu5 DVR and clinical characteristics.

In the first step, we determined mGlu5 DVR for 35 brain regions. We used the signal from the late imaging frames that capture a dynamic equilibrium (45–60 min). For each region, mGlu5 DVR was calculated as a ratio of the signal from the region of interest and the signal from the cerebellum as a reference region. For this step we used a maximum probability atlas and a segmentation procedure implemented in the PMOD PNEURO-Tool (PMOD Technologies, Switzerland, www.pmod.com). Data processing underwent visual inspection. The mGlu5 DVR-values from the first step were carried forward to the second step of statistical analysis, in which we compared mGlu5 DVR between both groups.

In the second step of statistical analyses, we compared mGlu5 DVR between both groups in each region with two-tailed Welch’s tests, uncorrected for multiple comparisons. These analyses were complemented by analyses of variance (ANOVAs) with mGlu5 DVR as the dependent variable, the factors “group” (BN vs. HC), “smoking” (smoker vs. non-smoker), and an interaction effect group*smoking. Importantly, in these analyses and the correlation analyses described in the following we grouped non-smokers and long-time ex-smokers together and labelled them as “non-smokers” (n = 9 in each diagnostic group). Furthermore, we grouped current smokers and short-time ex-smokers and labelled them as “smokers” (n = 6 in each diagnostic group). The rationale for this smoking subgroup-assignment was twofold. First, from a statistical point of view, a factor “smoking” with four levels (smoker, long-term ex-smoker, short-term ex-smoker, and non-smoker) and a sample size of n = 1 at some factor levels would not allow robust ANOVA model fitting. Second, our previous research showed that long-term ex-smokers display mGlu5 DVR levels comparable to those of non-smokers, whereas mGlu5 DVR in short-term ex-smokers is similar to that observed in current smokers^18^. We carried out ANOVAs for all 35 brain regions without correction for multiple comparisons. Welch’s tests were calculated with the statistical computing software R, version 3.5.1 (R Core Team, R Foundation for Statistical Computing, Vienna, Austria, www.R-project.org). ANOVA model fitting was carried out as implemented in the R package “ez”, version 4.4-0. In the third step of statistical analyses we explored the relationship between mGlu5 DVR and clinical characteristics. We tested whether the correlation pattern of mGlu5 DVR across the brain differs between both groups, as previously described^17^. Briefly, we calculated for each diagnostic group and each smoking subgroup separately a matrix of Pearson’s r correlations between all brain regions, comprising 595 unique correlation pairs (Supplementary Figs. S1, S2, S4, S5). Then, we compared mGlu5 DVR correlations between healthy smokers and smokers with bulimia, as well as between healthy non-smokers and non-smokers with bulimia (n = 6 smokers and n = 9 non-smokers in each diagnostic group). When comparing correlations between both diagnostic groups we used Fisher’s r-to-z transformation and a significance threshold of p < 0.001, two-tailed, without correction for multiple comparisons^17^.

To assess the effects of antidepressant medication on mGlu5 DVR in subjects with BN, we ran further explorative analyses. First, we compared mGlu5 DVR in subjects with BN that did not receive antidepressant treatment at the time of scanning (n = 3) to subjects with BN receiving selective serotonin reuptake inhibitors or selective serotonin-norepinephrine reuptake inhibitors (SSRIs and SSNRIs, n = 11). We carried out comparisons for 35 brain regions with Welch’s tests and a significance threshold of p < 0.05, two-tailed, without correcting for multiple testing. Second, to exclude a confounding effect of smoking, we repeated these comparisons in smokers only (n = 3 off antidepressant medication and n = 3 receiving SSRIs or SSNRIs).

Finally, we explored the relation between mGlu5 in each brain region and each quantified clinical characteristic within subjects with bulimia (n = 15). In these explorative analyses, we carried out a z-standardization of mGlu5 DVR-values to account for systematic differences between smokers and non-smokers^16,18^. We z-standardized mGlu5 DVR in smokers (n = 6) and non-smokers (n = 9) separately and pooled the z-standardized mGlu5 DVR values together (n = 15). Then, we calculated correlations between pooled z-standardized mGlu5 DVR-values and demographic and questionnaire data. We correlated z-standardized pooled DVR values from 35 brain regions with the following 33 demographic and questionnaire variables: Age, BDI score, BAI score, five EDE-Q subscales, eleven EDI-2 subscales and the total EDI-2 score, items 13–18 from EDE-Q (directly assessing bulimia nervosa symptoms), years of education, age of onset, illness duration, imipramine equivalent, body mass index at scan, body mass index at follow-up, and the number of weeks with binge and purge episodes in the six months following PET. This procedure resulted in 1,155 explorative correlation tests performed without correction for multiple testing. In this series of explorative analyses, we considered Pearson’s r-correlations with p < 0.05, two-tailed, uncorrected for multiple comparisons, for relevant. We carried out all exploratory correlation analyses in R and used the packages “Hmisc” (version 4.1-1) and “cocor” (version 1.1-3) for correlation comparisons, the packages “lattice” (version 0.20-35), “corrgram” (version 1.13), and “corrplot” (version 0.84) for visualization of results.

## Supplementary information

Supplementary Information.

## Data Availability

The datasets generated and analysed during the current study are available from the corresponding author on reasonable request.
