# Supplementary material for: Metabotropic glutamate receptor 5 in bulimia nervosa
Source: Sci Rep. 2020 Apr 14;10:6374. doi: 10.1038/s41598-020-63389-7 (PMC7156702; doi:10.1038/s41598-020-63389-7)
Supplement: Supplementary file 1 — Supplementary Information. [file 41598_2020_63389_MOESM1_ESM.doc]

**Supplemental Information**

**Metabotropic glutamate receptor 5 in bulimia nervosa**

**Authors:**

Yoan Mihov, Dr. phil.^1,6^, Valerie Treyer^2^, Ph.D., Funda Akkus^1^, M.D., Erika Toman^3^, Dr. phil., Gabriella Milos^4^, M.D., Simon M. Ametamey^5^, Ph.D., Anass Johayem^2^, Ph.D., Ph.D., Gregor Hasler*^1,6^, M.D.

^1^Translational Research Center, University Hospital of Psychiatry and Psychotherapy, University of Bern, Switzerland

^2^Departement of Nuclear Medicine, University Hospital Zürich, University of Zürich, 8091 Zürich, Switzerland

^3^Competence Network for Eating Disorders, Forchstrasse 132, 8032 Zürich, Switzerland

^4^Department of Consultation-Liaison Psychiatry and Psychosomatic Medicine, University Hospital Zürich, Culmannstrasse 8, 8091 Zürich, Switzerland

^5^Center for Radiopharmaceutical Science of ETH, PSI, and USZ, Department of Chemistry and Applied Biosciences of ETH, 8093 Zürich, Switzerland

^6^Psychiatry Research Unit, University of Fribourg, Chemin du Cardinal-Journet 3, 1752 Villars-sur-Glâne, Switzerland

*** Corresponding author**

Gregor Hasler, MD

Psychiatry Research Unit

University of Fribourg

Chemin du Cardinal-Journet 3

1752 Villars-sur-Glâne

E-Mail: gregor.hasler@unifr.ch

Phone: +41 26 304 16 89

**Supplementary Table S1.**

The effect of SSRI/SSNRI treatment on [11C]ABP688 DVR in Bulimia nervosa

| **Brain region** | **p-value** | **t-value** |
| --- | --- | --- |
| Middle frontal gyrus | 0.146852 | 1.723845 |
| Precentral gyrus | 0.07415 | 2.12868 |
| Straight gyrus | 0.139085 | 1.837161 |
| Anterior orbital gyrus | 0.228007 | 1.537539 |
| Inferior frontal gyrus | 0.13321 | 1.90001 |
| Superior frontal gyrus | 0.090329 | 2.041087 |
| Medial orbital gyrus | 0.142295 | 1.855815 |
| Lateral orbital gyrus | 0.185778 | 1.785166 |
| Posterior orbital gyrus | 0.086544 | 2.327652 |
| Subgenual anterior cingulate gyrus | 0.617882 | 0.558367 |
| Subgenual prefrontal cortex | 0.371518 | 1.017032 |
| Hippocampus | 0.523054 | 0.66116 |
| Amygdala | 0.232491 | 1.373674 |
| Anterior temporal lobe, medial part | 0.17209 | 1.704423 |
| Anterior temporal lobe, lateral part | 0.207101 | 1.628881 |
| Parahippocampal and ambient gyri | 0.126824 | 2.052777 |
| Superior temporal gyrus, posterior part | 0.100723 | 2.098204 |
| Middle and inferior temporal gyrus | 0.159712 | 1.764482 |
| Fusiform gyrus | 0.064595 | 2.499506 |
| Posterior temporal lobe | 0.122457 | 1.998386 |
| Superior temporal gyrus, anterior part | 0.294974 | 1.216677 |
| Postcentral gyrus | 0.111949 | 1.967687 |
| Superior parietal gyrus | 0.047009 | 2.563258 |
| Inferiolateral remainder of parietal lobe | 0.147539 | 1.825911 |
| Lateral remainder of occipital lobe | 0.072713 | 2.361648 |
| Lingual gyrus | 0.015142 | 3.194197 |
| Cuneus | 0.136198 | 1.959641 |
| Caudate nucleus | 0.104021 | 2.147965 |
| Nucleus accumbens | 0.016699 | 3.171868 |
| Putamen | 0.110713 | 2.106242 |
| Thalamus | 0.397711 | 1.005433 |
| Pallidum | 0.002316 | 3.900089 |
| Insula | 0.014537 | 3.259062 |
| Cingulate gyrus (gyrus cinguli), anterior part | 0.017824 | 3.177232 |
| Cingulate gyrus (gyrus cinguli), posterior part | 0.027879 | 3.147642 |

The table shows comparisons between [11C]ABP688 DVR in n = 11 subjects that received treatment with SSRIs or SSNRIs and n = 3 subjects that did not receive antidepressant treatment at the time of scanning. Comparisons were carried out with two-tailed Welch’s tests for 35 ***brain regions***. Reported ***p-values*** represent the results for two-tailed Welch’s tests without correction for multiple comparisons. Positive ***t-values*** indicate higher mean [11C]ABP688 DVR in subjects receiving antidepressant treatment than in subjects not receiving antidepressant medication. All three subjects without antidepressant medication were smokers. Three out of eleven subjects receiving antidepressant medication were smokers.

**Supplementary Table S2.**

**The effect of SSRI/SSNRI treatment on [11C]ABP688 DVR in smokers with Bulimia nervosa**

| **Brain region** | **p-values** | **t-values** |
| --- | --- | --- |
| Middle frontal gyrus | 0.359995 | 1.072432 |
| Precentral gyrus | 0.372485 | 1.066174 |
| Straight gyrus | 0.462288 | 0.830231 |
| Anterior orbital gyrus | 0.401244 | 0.948416 |
| Inferior frontal gyrus | 0.326753 | 1.143059 |
| Superior frontal gyrus | 0.334472 | 1.161173 |
| Medial orbital gyrus | 0.30832 | 1.174956 |
| Lateral orbital gyrus | 0.263575 | 1.327 |
| Posterior orbital gyrus | 0.250044 | 1.366999 |
| Subgenual anterior cingulate gyrus | 0.671628 | 0.459744 |
| Subgenual prefrontal cortex | 0.674577 | 0.463218 |
| Hippocampus | 0.271277 | 1.355599 |
| Amygdala | 0.457179 | 0.843971 |
| Anterior temporal lobe, medial part | 0.411645 | 0.93438 |
| Anterior temporal lobe, lateral part | 0.315622 | 1.153196 |
| Parahippocampal and ambient gyri | 0.2082 | 1.504868 |
| Superior temporal gyrus, posterior part | 0.385892 | 1.006209 |
| Middle and inferior temporal gyrus | 0.414414 | 0.93186 |
| Fusiform gyrus | 0.212627 | 1.524121 |
| Posterior temporal lobe | 0.343231 | 1.097244 |
| Superior temporal gyrus, anterior part | 0.585337 | 0.608073 |
| Postcentral gyrus | 0.347197 | 1.110449 |
| Superior parietal gyrus | 0.281 | 1.313452 |
| Inferiolateral remainder of parietal lobe | 0.358579 | 1.066751 |
| Lateral remainder of occipital lobe | 0.301851 | 1.226164 |
| Lingual gyrus | 0.111945 | 2.117607 |
| Cuneus | 0.29519 | 1.216825 |
| Caudate nucleus | 0.070509 | 2.450432 |
| Nucleus accumbens | 0.221669 | 1.586895 |
| Putamen | 0.293422 | 1.23005 |
| Thalamus | 0.223813 | 1.478968 |
| Pallidum | 0.304324 | 1.321264 |
| Insula | 0.290628 | 1.331753 |
| Cingulate gyrus (gyrus cinguli), anterior part | 0.267182 | 1.395408 |
| Cingulate gyrus (gyrus cinguli), posterior part | 0.160937 | 1.783682 |

The table shows comparisons between [11C]ABP688 DVR in n = 3 subjects that received treatment with SSRIs or SSNRIs and n = 3 subjects that did not receive antidepressant treatment at the time of scanning. All subjects were smokers. Comparisons were carried out with two-tailed Welch’s tests for 35 ***brain regions***. Reported ***p-values*** represent the results for two-tailed Welch’s tests without correction for multiple comparisons. Positive ***t-values*** indicate higher mean [11C]ABP688 DVR in subjects receiving antidepressant treatment than in subjects not receiving antidepressant medication.

**Supplementary Table S3.** **Effect sizes and statistical power for t-tests.**

| **Brain region** | **p-value** | **t-value** | **Cohen’s d** | **Power** | **HC mean** | **HC stdev** | **BN mean** | **BN stdev** |
| --- | --- | --- | --- | --- | --- | --- | --- | --- |
| **Middle frontal gyrus** | 0.127468548 | 1.571113639 | 0.573689587 | 0.329276328 | 1.433759432 | 0.179311876 | 1.541253527 | 0.195101865 |
| **Precentral gyrus** | 0.132348327 | 1.550111581 | 0.56602072 | 0.321997028 | 1.394212371 | 0.169281421 | 1.490846639 | 0.17215781 |
| **Straight gyrus** | 0.045811535 | 2.090443459 | 0.763322025 | 0.5233725 | 1.611564334 | 0.266966257 | 1.809495951 | 0.251406064 |
| **Anterior orbital gyrus** | 0.112681553 | 1.637694384 | 0.598001438 | 0.352783799 | 1.424584887 | 0.221811693 | 1.555855877 | 0.2171964 |
| **Inferior frontal gyrus** | 0.079959327 | 1.816859296 | 0.663423213 | 0.418713981 | 1.468400078 | 0.190392513 | 1.595426951 | 0.192545147 |
| **Superior frontal gyrus** | 0.124695835 | 1.582915494 | 0.577999015 | 0.33339641 | 1.445289365 | 0.191544594 | 1.558649996 | 0.200602788 |
| **Medial orbital gyrus** | 0.056856743 | 1.986570298 | 0.72539291 | 0.483426991 | 1.50517449 | 0.218658008 | 1.667534771 | 0.228873251 |
| **Lateral orbital gyrus** | 0.095787469 | 1.724241553 | 0.629603995 | 0.384208529 | 1.408331133 | 0.25936496 | 1.56443848 | 0.235973632 |
| **Posterior orbital gyrus** | 0.154862841 | 1.462090295 | 0.53387989 | 0.292272089 | 1.463736677 | 0.209833016 | 1.577684247 | 0.216973199 |
| **Subgenual anterior cingulate gyrus** | 0.173271523 | 1.397492355 | 0.510292058 | 0.271330985 | 1.589099033 | 0.242270744 | 1.716115305 | 0.255374706 |
| **Subgenual prefrontal cortex** | 0.045651122 | 2.092805373 | 0.764184474 | 0.524279631 | 1.694996752 | 0.248635886 | 1.89569996 | 0.275928809 |
| **Hippocampus** | 0.231584464 | 1.223037168 | 0.446590031 | 0.218942511 | 1.427300048 | 0.17039814 | 1.506894322 | 0.185725598 |
| **Amygdala** | 0.058851374 | 1.976236926 | 0.721619696 | 0.479455956 | 1.421174577 | 0.196798208 | 1.54662112 | 0.147347633 |
| **Anterior temporal lobe, medial part** | 0.165276417 | 1.424834476 | 0.520275989 | 0.280099526 | 1.514346684 | 0.22946166 | 1.631283028 | 0.219954379 |
| **Anterior temporal lobe, lateral part** | 0.06886488 | 1.89241065 | 0.691010667 | 0.44735441 | 1.512786924 | 0.251095802 | 1.680746836 | 0.234757851 |
| **Parahippocampal and ambient gyri** | 0.083972481 | 1.791868933 | 0.654298023 | 0.409328436 | 1.404139491 | 0.195377688 | 1.53326313 | 0.199296511 |
| **Superior temporal gyrus, posterior part** | 0.053076473 | 2.02066211 | 0.737841479 | 0.496538116 | 1.485788317 | 0.191172185 | 1.634745358 | 0.212051926 |
| **Middle and inferior temporal gyrus** | 0.06382916 | 1.929728613 | 0.704637261 | 0.46161488 | 1.499477729 | 0.221903102 | 1.655773871 | 0.221718424 |
| **Fusiform gyrus** | 0.06401797 | 1.928334906 | 0.704128351 | 0.461081273 | 1.54547385 | 0.247725225 | 1.717474272 | 0.240773812 |
| **Posterior temporal lobe** | 0.108469819 | 1.658076285 | 0.605443856 | 0.360101847 | 1.459598714 | 0.180401245 | 1.56994211 | 0.184084287 |
| **Superior temporal gyrus, anterior part** | 0.119769725 | 1.604792172 | 0.585987249 | 0.341088091 | 1.455819077 | 0.198413153 | 1.573745856 | 0.20403679 |
| **Postcentral gyrus** | 0.17810546 | 1.381353094 | 0.504398833 | 0.266222685 | 1.380458307 | 0.163203234 | 1.464068432 | 0.16828173 |
| **Superior parietal gyrus** | 0.305681738 | 1.043455309 | 0.381016007 | 0.172043818 | 1.399396356 | 0.153834724 | 1.459400173 | 0.161050055 |
| **Inferiolateral remainder of parietal lobe** | 0.194435051 | 1.329589368 | 0.485497393 | 0.250187864 | 1.442115266 | 0.174365772 | 1.529836705 | 0.186787948 |
| **Lateral remainder of occipital lobe** | 0.117485866 | 1.615185179 | 0.589782238 | 0.344766286 | 1.38439467 | 0.148069576 | 1.471242555 | 0.146434192 |
| **Lingual gyrus** | 0.246992239 | 1.182403565 | 0.431752736 | 0.207681658 | 1.401907214 | 0.138034406 | 1.462004328 | 0.140342742 |
| **Cuneus** | 0.226174971 | 1.237571249 | 0.451897126 | 0.223059793 | 1.440181023 | 0.139284507 | 1.504636378 | 0.145904284 |
| **Caudate nucleus** | 0.289676073 | 1.079311507 | 0.394108839 | 0.180807272 | 1.580371591 | 0.258395008 | 1.680068904 | 0.247423995 |
| **Nucleus accumbens** | 0.063384076 | 1.934116324 | 0.706239426 | 0.463295256 | 1.600649181 | 0.282345436 | 1.790058503 | 0.253253466 |
| **Putamen** | 0.208766343 | 1.287887718 | 0.470270103 | 0.237670047 | 1.559257543 | 0.245522812 | 1.664440216 | 0.199424325 |
| **Thalamus** | 0.824454343 | 0.223916592 | 0.081762779 | 0.055378741 | 1.239674225 | 0.116421158 | 1.249505618 | 0.123946858 |
| **Pallidum** | 0.15119624 | 1.476048774 | 0.538976806 | 0.296897424 | 1.092996218 | 0.122139891 | 1.162322121 | 0.13479851 |
| **Insula** | 0.089338728 | 1.761167585 | 0.643087476 | 0.397870617 | 1.490436153 | 0.204500023 | 1.632143694 | 0.235143346 |
| **Cingulate gyrus, anterior part** | 0.02740644 | 2.330004495 | 0.850797347 | 0.613964657 | 1.558320072 | 0.235051518 | 1.774800982 | 0.272461072 |
| **Cingulate gyrus, posterior part** | 0.083782972 | 1.795584054 | 0.655654594 | 0.410720509 | 1.482072438 | 0.191287045 | 1.622071745 | 0.233657821 |

***p-values*** and ***t-values*** refer to two-tailed Welch’s tests without correction for multiple comparisons (n = 15 per group); ***Cohen’s d***, as calculated with the package “effsize” in R; ***Power*** indicates statistical power (1 - β) for t-tests, as calculated with the package “pwr” in R; ***mean*** indicates average and ***stdev*** indicates standard deviation in healthy controls (***HC***) and subjects with bulimia (***BN***), as calculated in R.

**Supplementary Figure S1**

The figure shows a correlation matrix of mGlu5 DVR for 35 brain regions in healthy non-smokers (n = 9). We calculated the correlations using Pearson’s r, as previously reported ^1^. Brain regions are listed on the left and above the matrix. The colour indicates the value of Pearson’s r, as shown in the colour bar on the right. Thus, red tones indicate positive correlations, blue tones indicate negative correlations, and white indicates a correlation of zero.

**Supplementary Figure S2**

A matrix of mGlu5 DVR-correlations for 35 brain regions in non-smoking subjects with BN (n = 9). Annotation analogous to Supplementary Figure S1 ^1^.

**Supplementary Figure S3**

The figure shows numerical differences in mGlu5 DVR correlations between healthy non-smokers and non-smokers with bulimia nervosa (n = 9 per diagnostic group). We calculated and plotted the differences as reported earlier ^1^. Here, the colour represents the numerical difference ***Pearson’s*** ***r_BN_ - Pearson’s r_HC_***, as shown in the colour palette on the right. Thus, blue hues indicate higher correlations in HC, than in BN, red hues indicate higher correlations in BN, than in HC, and white squares correspond to a zero or a negligible difference.

**Supplementary Figure S4**

The figure shows a matrix of mGlu5 DVR Pearson’s r correlations in healthy smokers (n = 6). Annotation analogous to Supplementary Figure S1.

**Supplementary Figure S5**

The figure shows a matrix of mGlu5 DVR Pearson’s r correlations in smokers with BN (n = 6). Annotation analogous to Supplementary Figure S1.

**Supplementary Figure S6**

The figure shows numerical differences in mGlu5 DVR Pearson’s r correlations between healthy smokers and smokers with bulimia nervosa (n = 6 per diagnostic group). Annotation analogous to Supplementary Figure S3.

**Supplementary Figure S7**

Relationship between mGlu5 DVR in various brain regions (x-axes) and EDI-2 “Maturity fears” scores (y-axes) in subjects with bulimia nervosa (n = 15). mGlu5 DVR-values were z-standardized in smokers (n = 6) and non-smokers (n = 9) separately and pooled together for correlation analyses. For all shown relationships Pearson’s product-moment correlation test yielded p < 0.05, two-tailed, uncorrected for multiple testing.

**References**

1. Akkus, *F. et a*l. Metabotropic glutamate receptor 5 binding in male patients with alcohol use disorder*. Translational psychiatr*y**.** 8, 17, doi:10.1038/s41398-017-0066-6 (2018).
